# Genetic mapping and comparative genomics to inform restoration enhancement and culture of southern flounder, *Paralichthys lethostigma*

**DOI:** 10.1186/s12864-018-4541-0

**Published:** 2018-02-23

**Authors:** Shannon J. O’Leary, Christopher M. Hollenbeck, Robert R. Vega, John R. Gold, David S. Portnoy

**Affiliations:** 1 Department of Life Sciences, Marine Genomics Laboratory, Texas A&M University Corpus Christi, 6300 Ocean Drive, Unit 5869, Corpus Christi, TX 78412 USA; 2 Scottish Oceans Institute, University of St. Andrews, East Sands, St. Andrews, Fife, KY16 8LB UK; 3 Texas Parks and Wildlife Department, CCA Marine Development Center, 4300 Waldron Road, Corpus Christi, TX 78418 USA

**Keywords:** Linkage map, RADseq, Aquaculture, Synteny

## Abstract

**Background:**

Southern flounder, *Paralichthys lethostigma*, historically support a substantial fishery along the Atlantic and Gulf coasts of the southern United States. Low year-class strengths over the past few years in the western Gulf of Mexico have raised concern that spawning stocks may be overfished. Current management of the resource includes releasing hatchery-raised juveniles to restock bays and estuaries; additionally, there is a growing interest in the potential for commercial aquaculture of the species. Currently, genomic resources for southern flounder do not exist. Here, we used two hatchery-reared families and double-digest, restriction-site-associated DNA (ddRAD) sequencing to create a reduced-representation genomic library consisting of several thousand single nucleotide polymorphisms (SNPs) located throughout the genome.

**Results:**

The relative position of each SNP-containing locus was determined to create a high-density genetic map spanning the 24 linkage groups of the southern flounder genome. The consensus map was used to identify regions of shared synteny between southern flounder and seven other fish species for which genome assemblies are available. Finally, syntenic blocks were used to localize genes identified from transcripts in European flounder as potentially being involved in ecotoxicological and osmoregulatory responses, as well as QTLs associated with growth and disease resistance in Japanese flounder, on the southern flounder linkage map.

**Conclusions:**

The information provided by the linkage map will enrich restoration efforts by providing a foundation for interpreting spatial genetic variation within the species, ultimately furthering an understanding of the adaptive potential and resilience of southern flounder to future changes in local environmental conditions. Further, the map will facilitate the use of genetic markers to enhance restoration and commercial aquaculture.

**Electronic supplementary material:**

The online version of this article (10.1186/s12864-018-4541-0) contains supplementary material, which is available to authorized users.

## Background

Southern flounder, *Paralichthys lethostigma,* is a left-eyed, large-tooth flounder inhabiting bays and estuaries along the Atlantic coast of the United States from the Carolinas south through Florida and across the northern Gulf of Mexico (Gulf) to Tuxpan, Mexico; the species is notably absent along the southern Florida peninsula [1, 2]. The species is fished recreationally and commercially throughout its range [3, 4], though recent declines in abundance and low year-class strengths in the western Gulf have resulted in a growing interest in aquaculture for stock augmentation [3, 5]. Current management of the resource includes spawning wild-caught adults, rearing fingerlings in hatcheries, and releasing juveniles to augment natural recruitment [6, 7]. It is possible, given the species’ life history, that differences in environmental conditions (e.g., temperature, salinity) across estuaries and bays inhabited by southern flounder [8, 9] may have resulted in localized adaptation, in which case offspring from wild-caught adults adapted to those conditions would need to be released into specific localities to maximize efficiency of restocking [6]. In addition, given the species popularity as a food fish there is interest in the potential for commercial aquaculture [10].

Genomic resources can be useful in ensuring success of stock enhancement and commercial aquaculture [11]. Developments in sequencing and computational power have increased availability of genomic resources (e.g., whole-genome assemblies, transcriptomes) and furthered our understanding of the underlying genetic and physiological mechanisms controlling desirable traits, including genotype-environment interactions [11–13]. These resources also have facilitated marker-selected breeding to enhance traits such as growth rate, survivorship, and resistance to disease and/or parasites [14]. However, for most species, whole-genome assembly remains costly, time-consuming, and challenging. Alternatively, a highly dense linkage map, where genetic markers are localized onto individual linkage groups (chromosomes) and relative positions of the markers are summarized, can fulfill many of the purposes of a fully assembled and annotated genome by giving insight into genome structure and organization and further providing a resource for comparative bioinformatics [12, 14].

Sequencing of reduced representation libraries, using restriction-site associated DNA sequencing (RAD*seq*), is an ideal approach for creating linkage maps because it allows for simultaneous discovery and genotyping of several thousand, single nucleotide polymorphisms (SNPs) across multiple individuals [15–17]. As a result, high-density maps can be generated efficiently for a large number of markers and without the initial marker characterization required for microsatellites and expressed sequence tags [18]. The greater density of a SNP-based linkage map also increases the probability that loci associated with traits of interest can be identified [19–21]. Last, highly dense linkage maps can be used as scaffolds for genome assembles [22], to provide context for population and evolutionary genomic studies [23, 24], and to aid in comparative genomics in other, non-model species [25].

Comparative genomics approaches are important tools to enhance aquaculture for commercial and restorative purposes because they allow transfer of results from whole genome sequencing, transcriptome sequencing, and/or gene expression studies of model organisms to studies involving non-model species of economic importance [13]. One way to integrate genomic resources across species is based on identifying syntenic blocks, defined here as blocks of loci (genes, SNPs, other loci) on a single linkage group (or chromosome) found in the same order and uninterrupted by other shared markers on the genome of a species of comparison [26, 27]. Genes (or regions) of interest previously characterized in parallel studies of the same species, related species and/or model organisms can be putatively identified and localized in species of interest by locating the gene's position within a syntenic block in a linkage map using synteny mapping [18].

Here, we created a high-density consensus linkage map for southern flounder, consisting of 2847 SNP-containing loci spread over the 24 linkage groups and used the map to identify regions (blocks) of shared synteny between southern flounder and seven fish species for which chromosome-level genome assemblies are available. The identified syntenic blocks were then used to map microsatellites used to identify QTLs for growth and disease/parasite resistance in Japanese flounder [28–31] and transcripts that were differentially expressed in European flounder in response to changes in levels of salinity [32] and exposure to anthropogenic environmental pollutants, hereafter ‘pollutants’ [33], onto the southern flounder linkage map.

## Methods

### Reference and library construction, SNP filtering and genotyping

A reduced-representation reference genome comprised of DNA extracted from 24 wild-caught southern flounder was assembled using sequence data from a ddRAD (double digest RAD) library generated following [34]. The library was sequenced using an Illumina MiSeq DNA sequencer that produces 300 bp-long, paired-end reads which, when used to assemble a reduced representation reference genome, increases mapping efficiency during SNP calling [18]. Raw reads were demultiplexed using *process_radtags* [35], and reference contiguous sequence alignments (contigs) were reconstructed using the overlapping read (OL) assembly option in the *dDocent* pipeline [36] for c = 0.88, K_1_ = 2, and K_2_ = 1.

DNA was extracted from parents and offspring of two outbred crosses (185 and 175 progeny, respectively) reared at the CCA Marine Development Center (Texas Parks and Wildlife Department) in Corpus Christi, TX, using Mag-Bind Blood and Tissue DNA kits (Omega Bio-Tek). For each mapping cross, a male and female were strip-spawned. Fertilized eggs/larvae were reared in two separate tanks with standard light/dark cycle, temperature and access to food until they reached approximately 5–10 mm in length at which point they were removed from the tanks and placed in individual tubes with DMSO. CCA Marine Development Center routinely rears southern flounder for augmentation purposes and details of husbandry, spawning and collection procedures adhere to standard operating procedures for Texas Parks and Wildlife Department hatcheries [37, 38].

One ddRAD library was constructed per mapping family, following [34], and sequenced on a single lane of an Illumina HiSeq 4000 DNA sequencer (paired-end, 150 bp reads). Raw sequences were demultiplexed using *process_radtags* and quality trimmed. Read mapping and SNP calling were performed for each mapping cross, using the *dDocent* pipeline and the constructed reduced-representation reference genome. Raw SNPs were rigorously filtered using *VCFtools* [39]; contigs with a minimum sequence quality of 20, a minimum genotype call rate per locus of 90%, a minor allele count of 3, minimum depth of 3, mean minimum depth of 15, and a minimum minor allele frequency of 0.05 were retained, as were individuals with no more than 50% missing data. SNPs also were filtered based on allele balance, quality/depth ratio, mapping quality ratio of reference and alternate alleles, properly paired status, strand representation, and maximum depth. Next, complex polymorphisms were decomposed using *vcfallelicprimitives* [40] and indels removed from the data set. Finally, the program *rad_haplotyper*, [41] was used to collapse SNPs contained on the same contig into haplotypes. The resulting data set, consisting of SNP-containing loci (hereafter loci), was used for linkage map construction.

### Linkage map construction

For each mapping family *r/qtl* [42] was used to further filter the data set and create male- and female-specific linkage maps. *Onemap* [43] was used to generate family maps based on full-sibling genotypes; the two family maps were then merged using *LPmerge* [44] to create a single consensus map. For each of the four sex-specific maps, patterns of segregation distortion were assessed for each locus using a chi-square test as implemented in *r/qtl*; loci with significantly distorted segregation patterns at the 5% level, following Bonferroni correction for multiple comparisons, were removed from the data set. For sets of loci that had identical segregation patterns (i.e., no recombination events were observed between loci), only one locus was retained for mapping. Recombination fractions (rf) and log-odds (LOD) scores were calculated for each pairwise combination of the remaining loci; loci were then grouped into linkage groups based on a minimum LOD = 6 and a maximum rf = 0.35. After initial ordering, the quality of the loci, individuals, and locus order was assessed based on the presence of large gaps, excess numbers of crossovers per individual, and genotyping errors indicated by tight double-crossovers. After removing problematic loci and individuals, loci were re-ordered, and a sliding window used to compare alternate orders. The order with the lowest number of crossovers, highest likelihood, and resulting in the shortest chromosome was retained. The final order was reassessed based on the above parameters and sex-specific maps finalized by assigning all loci from co-segregating groups to the same location as the appropriate mapped locus.

Family maps were created for each mapping cross, using loci mapped in male- and/or female-specific maps. Again, for sets of loci that had identical segregation patterns, only one locus was retained for mapping. Two-point recombination fractions were calculated between all pairs of loci, using *onemap* which implements algorithms for simultaneous maximum-likelihood estimation of linkage and linkage phase in full-cross data sets [45]. Loci were assigned to individual linkage groups based on a minimum LOD = 6 and a maximum rf = 0.35, and ordered initially using a set of the most informative loci, i.e., loci for which segregation was tracked in both parents. The remaining loci (those for which segregation was observed in only one parent) were mapped by estimating the likelihood for all possible maps and placing each locus in the most likely position; alternative orders were compared using a ripple with a window size = 4. Family maps were finalized by assigning all loci from co-segregating groups to the same location as the appropriate mapped locus.

Before creating a consensus map, family maps were compared to identify incongruent ordering. Problematic loci always fell into a cluster of loci with zero observed recombination in at least one of the family maps. To resolve conflicts, these loci were removed from that family map. If a locus was in a cluster of loci with zero observed recombination in both maps, it was removed from the map with the larger cluster, and the remaining markers re-ordered and mapped. This process was repeated three times to ensure the best possible merging and ordering of loci, given the constraints of sample size and possible genotyping error. A total of 125 and 160 loci were removed from Family A and B maps, respectively, during this process to eliminate conflicts; they were not removed from the final consensus map. Finally, *LPmerge* was used to create a consensus linkage map by merging corresponding linkage groups from mapping crosses A and B, using linear programming to minimize the mean absolute error between the resulting consensus linkage map and the individual family maps.

### Comparative genomics and synteny mapping

The *synteny_mapper* pipeline [46] was used to determine patterns of synteny between the consensus linkage map and seven, chromosome-level genome assemblies representing Japanese flounder, *Paralichthys olivaceus,* European seabass, *Dicentrarchus labrax* (assembly accession no. GCA_000689215.1), barramundi, *Lates calcarifer* (assembly accession no. GCA_001640805.1), three-spined stickleback, *Gasterosteus aculatus* (assembly accession no. GCA_000180675.1), Nile tilapia, (GCA_001858045.2) *Oreochromis niloticus* (assembly accession no. GCA_001858045.2), fugu, *Takifugu rubripes* (assembly accession no. GCA_000180615.2), and green spotted puffer, *Tetraodon nigroviridis* (assembly accession no. GCA_000180735.1), following [27]. This approach blasts sequences of mapped loci to fully sequenced fish genomes and determines their relative positions on the corresponding chromosomes in order to identify syntenic blocks [18, 27]. Order mismatches separated by less than 5% of the total length of the linkage group were considered the result of either small-scale, local rearrangements or ordering errors due to inherent uncertainty in the mapping process; such mismatches were ignored in the process of identifying syntenic blocks. Genomes were chosen based on the genome assembly level (chromosome-level) and quality (assessed using scaffold N50 and total assembly gap length) and included a variety of species (from species in the same genus, *Paralichthys* to more distantly related puffers), most of which have been successfully used in the past as comparison genomes for the *synteny_mapper.pl* pipeline [18, 27].

Two data sets containing sequences from expressed transcript studies of European flounder and three data sets from QTL studies of Japanese flounder were downloaded and synteny mapped as described in [27]. In brief, the downloaded sequences are blasted against all seven sequenced genomes to identify sequences falling into syntenic blocks. Those loci are then mapped onto the linkage map by identifying the SNP-containing loci flanking the sequence. The first data set from European flounder contained loci that were significantly up- or down-regulated in response to exposure to pollutants [32]; while the second data set contained loci that were expressed differentially in fish exposed to differences in salinity [33]. The QTL studies were based on microsatellite linkage maps developed for Japanese flounder [28, 29] and focused on growth [28] and resistance to lymphocystis [30] and *Edwardsiella tarda* [31].

Linkage maps and other figures were generated using *ggplot2* [47] and *circos* [48]. An extended version of Materials and Methods, including details on bioinformatic processing steps and statistical analysis, is available in Additional file 1. R notebooks containing reproducible code are available in a GitHub repository at https://github.com/sjoleary/SFL_LinkageMap.

## Results

### Reference construction, genotyping and SNP filtering

The final reduced-representation reference genome consisted of 52,831 RAD fragments (mean length = 264 bp; mode = 302 bp) totaling 14 Mb. Based on the range (500–800 Mb) of genome sizes in three other species in the genus *Paralichthys* [49], 14 Mb represents approximately 1.5–2.5% of the southern flounder genome. The unfiltered SNP data sets consisted of 185 individuals genotyped for 178,644 SNPs on 39,078 contigs (Family A) and 175 individuals genotyped for 447,144 SNPs on 39,950 contigs (Family B). The filtered SNP data sets consisted of 15,180 SNPs on 4461 contigs genotyped for 183 individuals (Family A) and 16,802 SNPs on 4357 contigs genotyped for 167 individuals (Family B). After haplotyping and further filtering, final data sets consisted of 2773 haplotyped loci (parents + 162 offspring, Family A) and 2353 haplotyped loci (parents + 152 offspring, Family B). SNP filtering steps and number of SNPs and contigs remaining at each step may be found in Additional file 2.

### Linkage map construction

The total number of SNP-containing loci mapped, total map length, mean number of loci, mean length per linkage group (LG), and mean distance between loci for linkage maps by family and by sex are summarized in Table 1. The merged consensus map consisted of 2847 loci spread across 24 linkage groups and a total length of 1,605.4 cM. Individual linkage groups ranged in number of loci (89–161; mean ± SE = 118.6 ± 18.8) and length (53.2–78.5 cM; mean ± SE = 66.9 ± 6.6), with a mean marker interval (overall) of 0.6 cM. A total of 1674 markers were mapped in Family A (1,303 and 1453 in female- and male-specific maps, respectively); while a total of 1305 markers were mapped in Family B (1,087, and 1078 in female- and male-specific maps, respectively). Individual loci and their position on each linkage group for all seven maps are summarized in Additional file 3; detailed information on number of loci per LG and LG lengths are summarized in Additional file 4.

**Table 1:** Summary statistics for consensus (Con) map, family maps (FamA, FamB) and sex-specific maps (female, ♀, male, ♂)

	Con	FamA	FamA ♀	FamA ♂	FamB	FamB ♀	FamB ♂
Total loci	2847	1674	1303	1453	1305	1087	1078
Total length [cM]	1,605.43	1,355.22	1,539.87	1,569.80	1,469.48	1,175.51	1,435.56
Mean ± SD loci per LG	118.6 ± 18.8	69.7 ± 12.7	54.3 ± 11.3	60.5 ± 11.9	54.4 ± 10.4	45.3 ± 12.5	44.9 ± 14.0
Mean ± SD LG size	66.9 ± 6.6	56.5 ± 9.2	64.2 ± 16.3	65.4 ± 8.1	61.2 ± 21.0	49.0 ± 13.1	59.8 ± 17.5
Mean locus interval	0.6	0.8	1.2	1.1	1.1	1.1	1.3

### Comparative genomics & synteny mapping

**Fig. 1:**
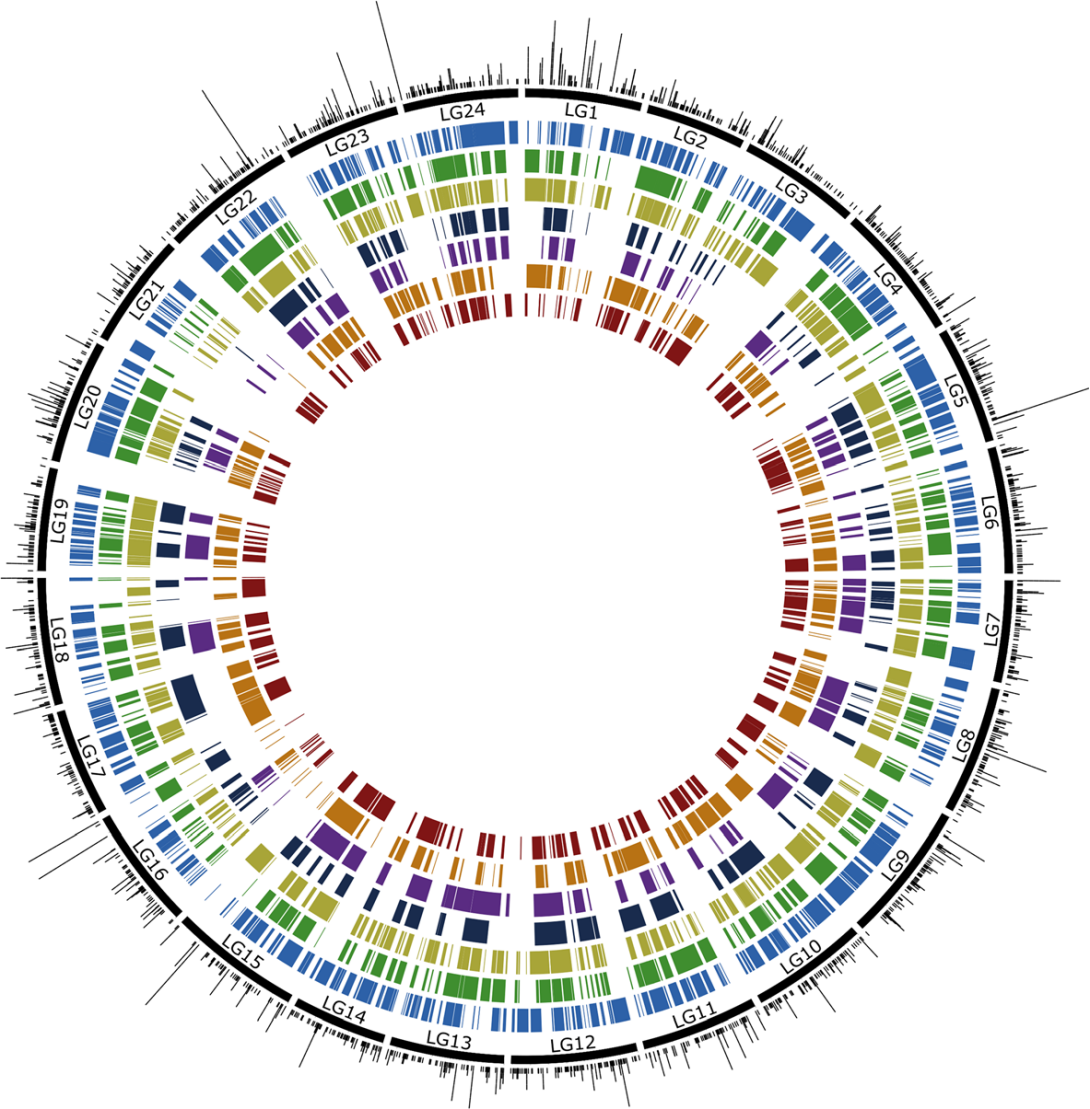
Circular ideogram depicting locations of identified syntenic blocks on the southern flounder consensus linkage map. Black rectangles represent southern flounder linkage groups. Black ticks on the outside indicate location of loci on the southern flounder linkage map; loci mapped to the same location are stacked. Colored segments on the inside represent syntenic blocks identified in comparisons of southern flounder sequences to genome sequences of Japanese flounder (blue), European seabass (green), barramundi (olive), fugu (dark blue), green spotted puffer (purple), nile tilapia (orange), and three-spined stickleback (red)

**Fig. 2:**
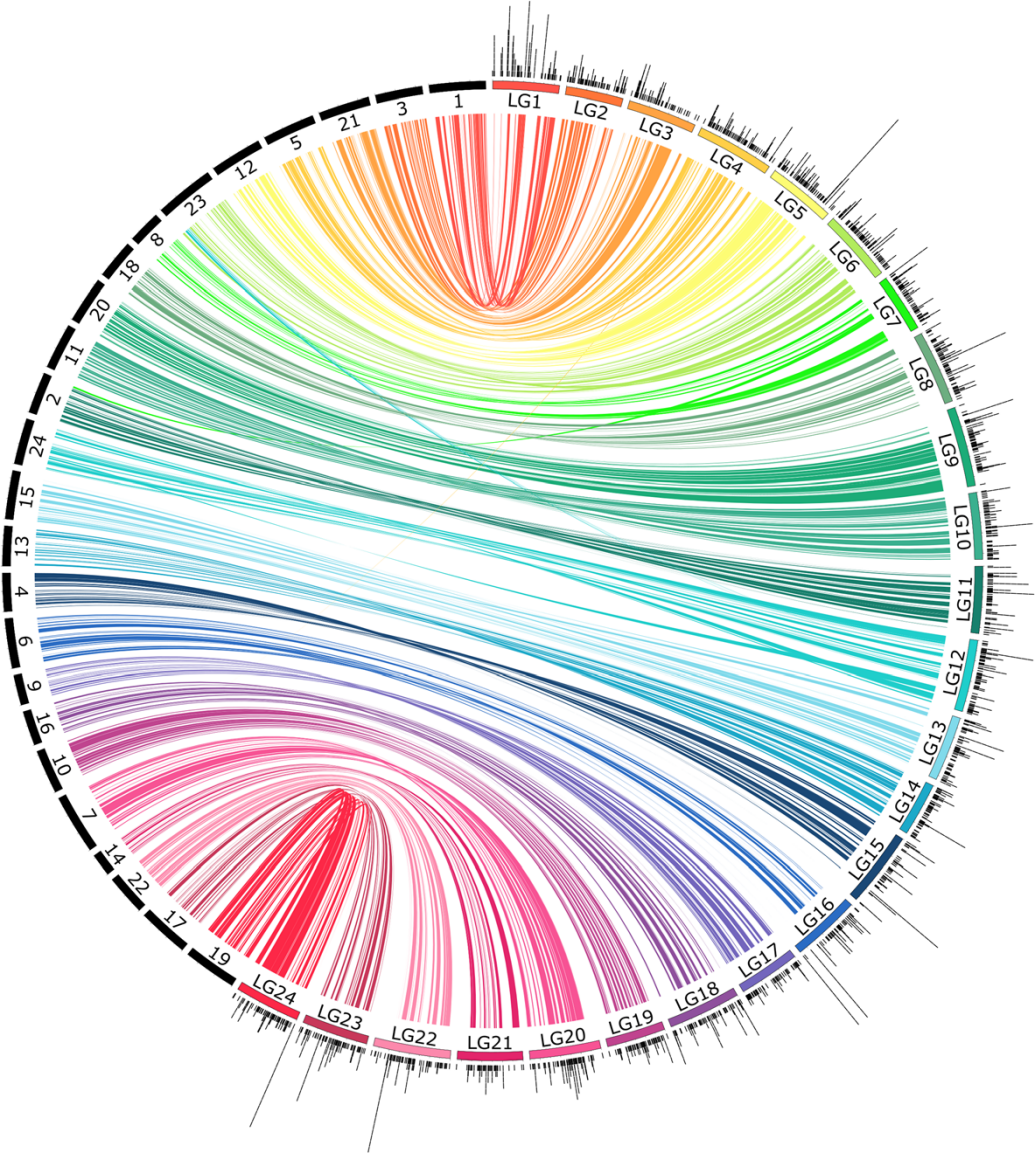
Comparative view of location of syntenic blocks on consensus linkage map of southern flounder and Japanese flounder. Solid black rectangles represent chromosomes of Japanese flounder. Black ticks indicate the positions of loci mapped on southern flounder linkage groups (colored rectangles); loci mapped to the same location are stacked. Syntenic blocks are connected by ribbons; the color corresponds to the color of each southern flounder linkage group. Width of the ribbon is proportional to the size of the syntenic block on a linkage group and its corresponding location on the chromosome of each comparison species

**Fig. 3:**
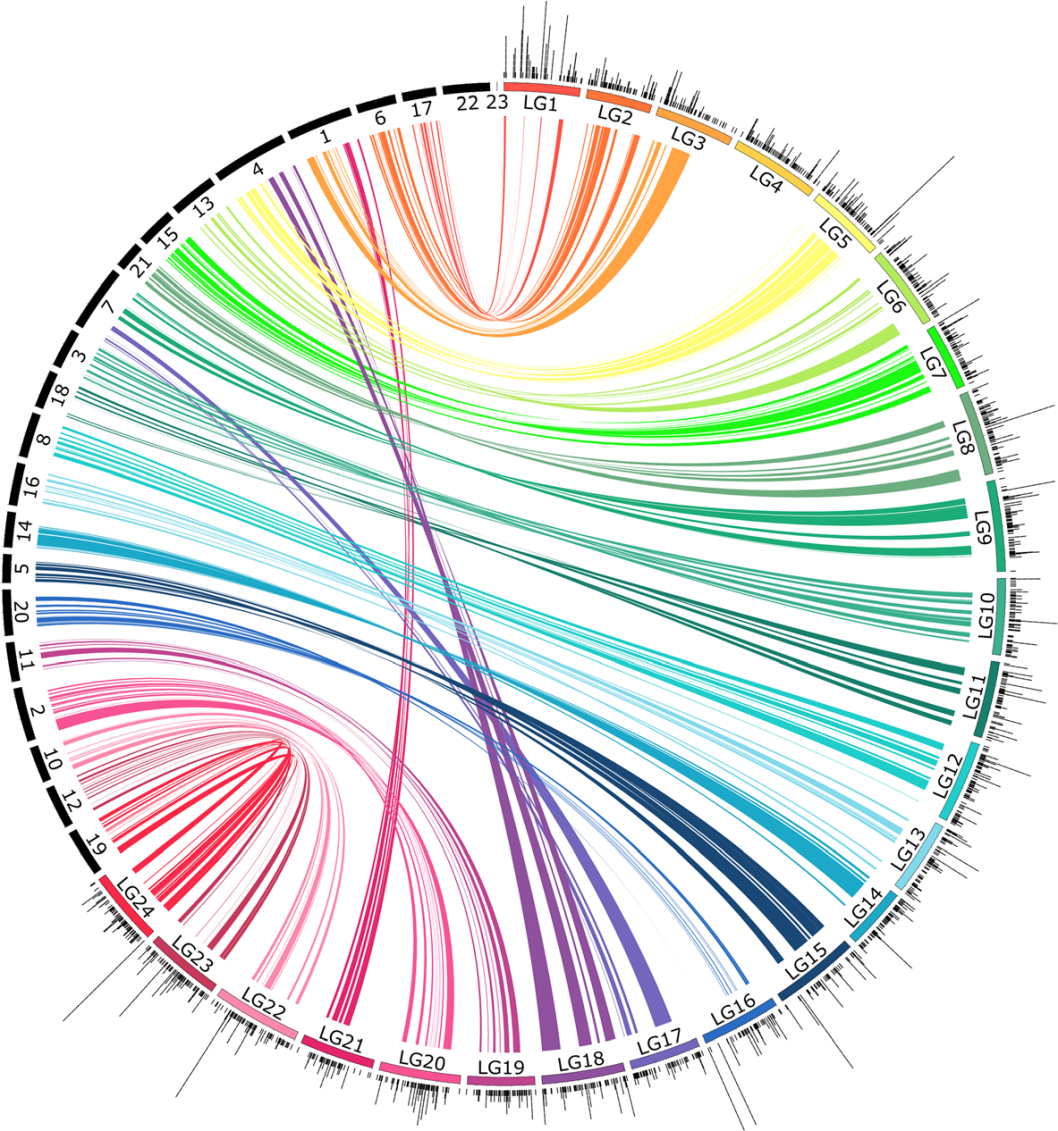
Comparative view of location of syntenic blocks on consensus linkage map of southern flounder and three-spined stickleback. Solid black rectangles represent chromosomes of three-spined stickleback. Black ticks indicate the positions of loci mapped on southern flounder linkage groups (colored rectangles); loci mapped to the same location on the linkage map are stacked. Syntenic blocks are connected by ribbons; the color corresponds to the color of each linkage group. Width of the ribbon represents size of the syntenic block on a linkage group and its corresponding location on the chromosome of each comparison species

Syntenic blocks identified between the southern flounder consensus map and each of the sequenced fish genomes are shown in Fig. 1; summary statistics for syntenic blocks are given in Table 2. The number of blocks identified ranged from 107 (green spotted puffer) to 511 (Japanese flounder) and the number of southern flounder loci contained across blocks ranged from 340 (green spotted puffer) to 1900 (Japanese flounder). Total block size (in Mb) ranged from 90.9 (green spotted puffer) to 287.5 (Japanese flounder), while average block size (in Mb/block) ranged from 1.22 (Nile tilapia) to 0.56 (Japanese flounder). Total block size (in cM) across blocks ranged from 511.7 (fugu) to 750.5 (Japanese flounder), while average block size (in cM) ranged from 1.47 (Japanese flounder) to 4.8 (green spotted puffer). The proportion of the southern flounder linkage map encompassed by the syntenic blocks ranged from 31.9% (fugu) to 46.7% in Japanese flounder. Summed across all seven fish genomes, approximately 83% of the southern flounder linkage map was covered by syntenic blocks, overall. The level of chromosomal rearrangement varied depending on species, with some species sharing the same number of chromosomes showing little to no rearrangement (e.g. Japanese flounder, Fig. 2) and other species exhibiting patterns of both inter- and intra-chromosomal rearrangement relative to southern flounder (e.g. three-spined stickleback, Fig. 3). Figures for other species are available as Additional files 5, 6, 7, 8 and 9.

**Table 2:** Summary statistics of syntenic blocks identified in seven fish genomes with the southern flounder linkage map

Species	Number of blocks/loci^a^	Average number of loci^a^/block	Total/average block size in Mbp	Total/average block size in cM	Proportion of linkage map covered
Japanese flounder	511/1,900	3.7	287.52/0.56	750.5/1.47	46.7
European seabass	295/1,100	3.7	271.67/0.92	700.5/2.3	43.6
Barramundi	360/1,302	3.6	283.49/0.78	720.0/2.0	44.9
Stickleback	195/626	3.2	157.78/0.80	556.7/3.8	34.7
Nile tilapia	219/764	3.4	267.13/1.22	612.1/2.7	38.1
Green spotted puffer	107/340	3.1	90.85/0.84	516.2/4.8	32.2
Fugu	142/420	2.9	108.49/0.76	511.7/3.6	31.9

^a^Loci on southern flounder consensus map

**Fig. 4:**
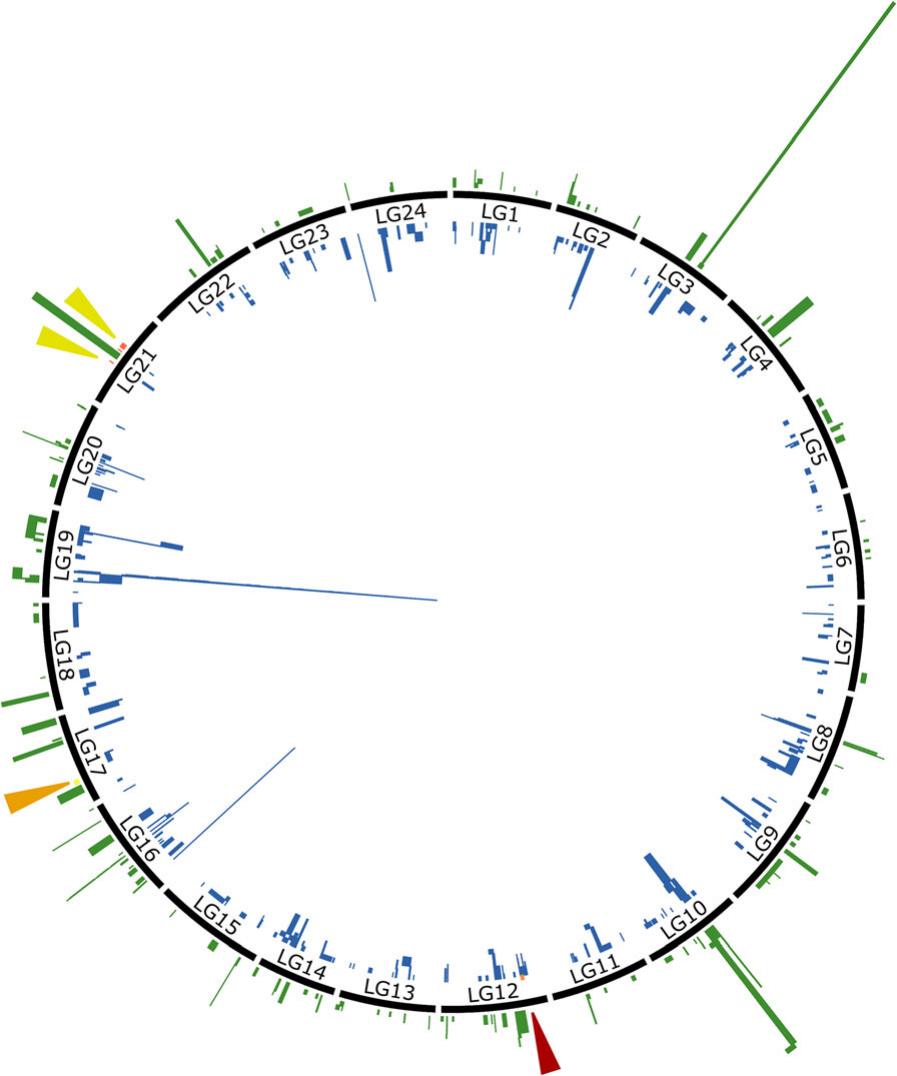
Circular ideogram showing locations of synteny mapped transcripts and QTLs. Black rectangles represent southern flounder linkage groups. The location of QTLs identified in Japanese flounder are indicated by red (QTL for resistance to lymphocystis [30]), orange (QTL for resistance to *Edwardsiella tarda* [31]), and yellow (QTLs for growth; qWi-f14–1, qWi-f14–2 and qWe-f14, [64]).Genes identified as being significantly up- or down-regulated in European flounder when exposed to pollutants [33] are represented in blue inside the circle; genes identified as being differentially expressed in the context of salinity differences [32] are indicated in green outside the circle. Relative height of bars represents number of transcripts mapped to a single location

Overall, 73% of the loci in the downloaded data set consisting of microsatellites and differentially expressed genes blasted against the seven genomes fell into synteny blocks and were then successfully mapped onto the southern flounder linkage map. The efficiency of the synteny mapping approached varied by genome from approximately 12% (green spotted puffer) to 67% (Japanese flounder) of loci being successfully located. Over 68% of loci fell into homologous synteny blocks on more than one genome. Of the loci mapped by only one genome, 48% were mapped using the Japanese flounder genome. A total of 1780 microsatellites from mapping studies of Japanese flounder [28, 29] were placed onto the southern flounder consensus map as were five QTLs (Fig. 4). A total of 1316 transcripts that were up- or down-regulated in European flounder when exposed to pollutants and to differences in salinity also were successfully localized on the consensus map (Fig. 4). Transcript locations were not evenly distributed among linkage groups and more than half of the transcripts were associated in clusters with at least one other transcript. Linkage groups 16 and 19 had the largest number of transcripts associated with exposure to pollutants (73 and 226, respectively); while linkage groups 3, 10, and 16 had the largest number of transcripts associated with differences in salinity (16, 16, and 19, respectively). Linkage group 19 had the largest number of clusters of transcripts (129) from the study of exposure to pollutants; while linkage group 3 had the largest number of clusters of transcripts (15) from the study of salinity differences. Transcripts from the study of exposure to pollutants that mapped to clusters of ten or more were identified as belonging to 19 genes, most prominently hemoglobin alpha and beta chains and the hepcidin/hepcidin-like precursor; gene ontology terms for mapped transcripts included haem/haemoglobins, immune function and inflammation, and phase I/II metabolism. Transcripts from the study of salinity differences that mapped to clusters with five or more loci were identified as belonging to eight genes, including apolipoprotein, prothrombin precursor, 60S ribosomal proteins, and glycoprotein; gene ontology terms for mapped transcripts included lipoprotein metabolism/lipid transport, protein biosynthesis/ribosome biogenesis and assembly, and blood coagulation proteolysis/peptidolysis. Details on genes and gene-ontology terms for all transcripts, including those not successfully synteny mapped, may be found in the original publications [32, 33]; synteny mapping information is available for download at https://github.com/sjoleary/SFL_LinkageMap/tree/master/results.

## Discussion

Comparative genomics is a powerful tool for transferring, integrating, and linking genomic information gathered from functional genomics, proteomics, and metabolomics between model and non-model species. For exploited and cultured non-model species, these approaches can be used to augment understanding of reproduction, growth, development, and disease resistance [11, 13, 50]. In this study, a total of 2847 haplotyped SNP-containing loci were mapped to 24 linkage groups that correspond to the 24 haploid chromosomes of southern flounder. The average mean locus interval was approximately 0.5 cM. Approximately 66% of the mapped loci were located on at least one of the seven assembled genomes of other fish species, enabling identification of regions (syntenic blocks) of shared synteny. A total of approximately 73% of the microsatellites and transcripts from studies of Japanese and European flounder, were successfully mapped onto the southern flounder linkage map using the identified synteny blocks.

Creating a linkage map consisting of RAD*seq*-derived, SNP-containing loci allows generating high-density linkage maps without requiring a separate marker development step, making the procedure more time- and cost-efficient than previous approaches, e.g. microsatellites or RFLPs. By using paired-end sequencing, sequences of approximately 300 bp in length were incorporated into the map; thus optimizing downstream comparative genomics [18]. To account for SNPs on the same contig, loci were haplotyped, allowing the information contained in each SNP on a single haplotype to be retained and reducing the number of markers incorporated in mapping analysis. Haplotyping also allowed detection and removal of potential multi-locus contigs (paralogs), which can result in conflicts in downstream marker ordering [18, 41].

In general, creating a consensus map from multiple mapping families increases the number of informative loci that can be successfully mapped [51], but comes at the cost of limiting the number of offspring that can be mapped per family, which determines the minumum frequency with which recombination needs to occur to be detected. In addition, using multiple families potentially introduces artifacts (e.g., inaccurate map lengths) due to differences in recombination rates among individuals, sexes or populations, to segmental duplicates, genotyping error, and/or differences in how well certain areas of the linkage map are reconstructed in different individuals and families [44, 52, 53]. Here, we chose to include two mapping crosses with at least 150 offspring, allowing us to map more loci (2847) than would have been possible with either cross alone, while at the same time retaining the ability to observe recombination at a rate as low as 0.16%. To deal with ordering conflicts, problematic loci were iteratively removed [54] if including them resulted in large gaps (> 20 cM) or removing them resulted in a significantly shorter linkage group and/or a higher LOD score [55]. While regions proximal to telomeres often tend to have more recombination in males, and regions proximal to centromeres frequently will have more observed recombination in females, causing ordering conflicts [56–58], we did not identify consistent, significant differences between the sexes. In general, the approach followed here was conservative because the eventual goal was to use the map in a comparative bioinformatic framework; consequently, we prioritized confidently identifying the relative position of fewer loci over inclusion of more loci and increased accuracy of map lengths [18, 27].

The effectiveness of the comparative bioinformatic framework applied here varied depending on the comparison genome. The highest coverage of synteny blocks (and the largest degree of conservation of the location of the synteny blocks) was found between southern flounder and Japanese Flounder (46.7%), European seabass (43.6%), and barramundi (44.9%). However, coverage of syntenic blocks across all comparison species was 83%, emphasizing a benefit of using multiple, assembled genomes for synteny mapping. The success of identifying blocks of synteny between genomes and a linkage map is likely a function of multiple factors: quality of the sequenced genome, density of the linkage map, size of genomes available for comparison, number of chromosomes in comparison species relative to the target species, and phylogenetic relationships of comparison species relative to one another. Aside from Japanese flounder (congeneric with southern flounder) European seabass and barramundi are related comparatively to flounders [59, 60], have a large amount of their genome sequenced (~ 580 Mb), and share the same number of haploid chromosomes (24) as southern flounder. Alternatively, the green spotted puffer, fugu, and Nile tilapia have a reduced number of chromosomes (21–22), which may explain why only 32–38% of the linkage map is represented in syntenic blocks on these genomes, with incongruent placement of some blocks due to chromosomal rearrangement.

Using synteny mapping, we localized five QTLs from Japanese flounder that were associated with resistance to viral/bacterial infection and to growth characters, as well as, transcripts from European flounder that were up and down regulated in response to a variety of anthropogenic pollutants found in the environment and to differences in salinity. Lymphocystis, a viral disease, has been found in wide range of cultured marine and freshwater fishes, including flounders [61, 62]. Identification of the QTL associated with lymphocystis resistance [30] quickly led to marker-assisted breeding for lymphocystis resistance in Japanese flounder [30]. Similarly, *Edwardseilla tarda*, an enteric bacteria, has been implicated in mortality of several fishes undergoing culture, including flatfishes [63]. The QTLs qWi-f14–1, qWi-f14–2, and qWe-f14 accounted for 16.75% and 13.62% of variation in body width, and 14.85% of variation in body weight in experiments with Japanese flounder [64]. All three map to southern flounder linkage group 21, (homolog to the Japanese flounder chromosome 14 on which the QTLs are located), suggesting that linkage group 21 may be an important target for studies of southern flounder growth characteristics. Because QTLs may vary across families and populations within species, comparative genomics approaches do not replace classic QTL and candidate gene experiments. Rather, they should be viewed as useful approaches for exploratory analyses *in silico* to guide experimental study design for characterizing genes that control traits of interest. Further, comparative approaches can be used to integrate and compare results of similar experiments performed in multiple species to understand shared genes/pathways and differences across taxa. Around 30% of all mapped transcripts from the studies of response to anthropogenic pollution and salinity changes in European flounder [32, 33] were localized to five large clusters, consistent with theory [65] and observations [66, 67] that co-adapted genes often are located in genomic clusters, sometimes referred to as ‘genomic islands of divergence’ [67]. Clusters identified on these linkage groups could serve as a starting point to design studies in southern flounder to understand genetic mechanisms of pollution resistance and salinity change both in wild and cultured populations.

## Conclusions

Our study demonstrates that genetic information useful to culture and management of fish species can be compared and integrated among species by using linkage maps and synteny mapping in combination with high quality chromosome-level genome assemblies. A substantial amount of genomic resources are now available for cultured flatfishes, including chromosome-level genome assemblies [68, 69], transcriptomes, gene expression, and QTL data [13], which can be mined for genetic sequences to experimental design for marker-assisted selection studies in southern flounder. Information gained from gene expression studies also will prove useful for understanding the potential for local adaptation. For southern flounder, this will strengthen the foundation for brood-selection and restocking, as well as provide insight into resilience to future climactic changes and anthropogenic disturbances.

## Additional files

Additional file 1: Detailed Methods & Materials. (DOCX 40 kb)

Additional file 2: Filtering parameters for each filtering step, and number of SNPs and contigs remaining for Families A and B. (DOCX 15 kb)

Additional file 3: Excel file containing a spreadsheet with locus, linkage group, and position for the consensus map, family map A and B, and sex-specific maps for male and females for each mapping family. (XLSX 251 kb)

Additional file 4: Comparison of number of loci and linkage group length (in parentheses) for corresponding linkage groups for consensus map (LG), family maps (LG_Fam A, LG_Fam B), and sex-specific maps (LG_Fam A_F, LG_FamA_M, LG_FamB_A, LG_FamB_M). (DOCX 16 kb)

Additional file 5: Comparative view of location of syntenic blocks on consensus linkage map of southern flounder and European seabass. Solid black rectangles represent chromosomes of European seabass. Black ticks indicate the positions of loci mapped on southern flounder linkage groups (colored rectangles); loci mapped to the same location are stacked. Syntenic blocks are connected by ribbons; the color corresponds to the color of each linkage group. Width of the ribbon represents size of the syntenic block on a linkage group and its corresponding location on the chromosome of each comparison species. (PNG 3116 kb)

Additional file 6: Comparative view of location of syntenic blocks on consensus linkage map of southern flounder and fugu. Solid black rectangles represent chromosomes of fugu. Black ticks indicate the positions of loci mapped on southern flounder linkage groups (colored rectangles); loci mapped to the same location are stacked. Syntenic blocks are connected by ribbons; the color corresponds to the color of each linkage group. Width of the ribbon represents size of the syntenic block on a linkage group and its corresponding location on the chromosome of each comparison species. (PNG 2296 kb)

Additional file 7: Comparative view of location of syntenic blocks on consensus linkage map of southern flounder and barramundi. Solid black rectangles represent chromosomes of barramundi. Black ticks indicate the positions of loci mapped on southern flounder linkage groups (colored rectangles); loci mapped to the same location are stacked. Syntenic blocks are connected by ribbons; the color corresponds to the color of each linkage group. Width of the ribbon represents size of the syntenic block on a linkage group and its corresponding location on the chromosome of each comparison species. (PNG 584 kb)

Additional file 8: Comparative view of location of syntenic blocks on consensus linkage map of southern flounder and nile tilapia. Solid black rectangles represent chromosomes of nile tilapia. Black ticks indicate the positions of loci mapped on southern flounder linkage groups (colored rectangles); loci mapped to the same location are stacked. Syntenic blocks are connected by ribbons; the color corresponds to the color of each linkage group. Width of the ribbon represents size of the syntenic block on a linkage group and its corresponding location on the chromosome of each comparison species. (PNG 2546 kb)

Additional file 9: Comparative view of location of syntenic blocks on consensus linkage map of southern flounder and green spotted puffer. Solid black rectangles represent chromosomes of green spotted puffer. Black ticks indicate the positions of loci mapped on southern flounder linkage groups (colored rectangles); loci mapped to the same location are stacked. Syntenic blocks are connected by ribbons; the color corresponds to the color of each linkage group. Width of the ribbon represents size of the syntenic block on a linkage group and its corresponding location on the chromosome of each comparison species. (PNG 1925 kb)

## Abbreviations

ddRAD: double digest restriction site-associated DNA
LG: Linkage group
LOD: Log-odds scores
QTL: Quantitative trait loci
RADseq: Restriction site-associated DNA sequencing
rf: Recombination frequency
SNP: Single-nucleotide polymorphism
