# Supplementary material for: Genetic mapping and comparative genomics to inform restoration enhancement and culture of southern flounder, Paralichthys lethostigma
Source: BMC Genomics. 2018 Feb 23;19:163. doi: 10.1186/s12864-018-4541-0 (PMC5824557; doi:10.1186/s12864-018-4541-0)
Supplement: Supplementary file 1 — Detailed Methods & Materials. (DOCX 40 kb) [file 12864_2018_4541_MOESM1_ESM.docx]

**Additional File 1**: Detailed Methods & Materials

## De novo reference construction

A reduced-representation reference genome was constructed using 24 southern flounder sampled from across the species’ geographic range and the four parents used for mapping crosses. A *dd*RAD library was constructed following Portnoy et al. [1] and sequenced on an Illumina MiSeq DNA sequencer producing 300 bp-long, paired-end reads. Raw reads were demultiplexed using *process_radtags* [2] and a reduced-representation reference genome assembled *de novo*, using the overlapping read (OL) assembly option in the *dDocent* pipeline [3]. Briefly, *dDocent* first uses *CD-HIT* [4] to cluster reads based on a user-selected level of %-similarity into putative loci. The user then selects cut-off values for within- and between-individual coverage (K_1_ and K_2_, respectively). Finally *dDocent* uses *PEAR* [5] to merge overlapping forward and reverse reads and cluster the merged reads, using *CD-HIT*; the longest sequence is selected for the reduced-representation reference. The %-similarity value was chosen after running *dDocent* for a range of c = 0.8 – 0.98 for K_1_ = 1 – 10 and K_2_ = 1-10 in order to identify the value for which a sudden increase in the number of loci in the reference is observed, indicating that loci are being over-split, i.e., alleles are separated into individual ‘loci’, thus creating artefactual contigs [6]. Values for K_1_ and K_2_ were chosen after mapping a set of 20 individuals to references generated for K_1_ and K_2_ = 1 – 4, using *BWA* [7], and comparing the number of reads mapped as proper pairs (i.e., both the forward and reverse read mapped to the same sequence in the reference genome) and as a mismatch (i.e., forward and reverse reads are mapped to two separate sequences). The final reduced-representation reference genome was constructed for c = 0.88, K_1_ = 2, and K_2_ = 1; details of reference construction can be found at in an R notebook at <https://github.com/sjoleary/SFL_LinkageMap/blob/master/Reference_Construction.Rmd>).

## Library construction, genotyping & SNP filtering

DNA was extracted using Mag-Bind Blood and Tissue DNA kits (Omega Bio-Tek) from tissue samples from parents and offspring of two outbred, full-sibling mapping crosses (185 and 175 progeny, respectively) reared at the CCA Marine Development Center (Texas Parks and Wildlife Department) in Corpus Christi, TX. One *dd*RAD library per mapping family was constructed as outlined in Portnoy *et al*. (2015) and sequenced on two lanes of an Illumina HiSeq 5000 DNA sequencer (paired-end, 150 bp reads). Raw sequences were demultiplexed using *process_radtags*. Demultiplexed sequences were quality trimmed using the *dDocent* pipeline and mapped to the reduced-representation reference genome constructed as described above. Read mapping, using *BWA* [7], and SNP calling, using *freebayes* [8], were performed separately for each mapping cross. Raw SNPs were rigorously filtered using *VCFtools* [9] and custom scripts. SNPs were filtered iteratively to maximize number of loci and individuals retained in the data set. The detailed filtering process is described in an R notebook available at https://github.com/sjoleary/SFL_LinkageMap/blob/master/Genotyping.Rmd. The final data set contained only SNPs with a minimum sequence quality of 20, a minimum genotype call-rate per locus of 90%, a minor allele count of 3, minimum depth of 3, mean minimum depth of 15, minimum minor allele frequency of 0.05, and individuals with no more than 50% missing data. Additionally, SNPs were filtered based on allele balance, quality/depth ratio, mapping quality ratio of reference and alternate alleles, properly paired status, strand representation, and maximum depth. Complex polymorphisms were then decomposed using *vcfallelicprimitives* [10] and indel sites removed from the data set (Additional File 2).

Finally, the program *rad_haplotyper* [11] was used to collapse SNPs contained on the same contig into haplotypes (Willis et al. 2017). In addition, the program was used to flag loci with an excess or deficit of detected haplotypes, as compared to SNP genotypes, to identify potential multi-locus contigs and/or genotyping errors, respectively. The haplotyped data set was then filtered to remove loci that had been flagged as potential multi-locus contigs in more than 5% of individuals, loci containing >5 SNPs and/or >4 haplotypes within a family, loci haplotyped in less than 95% of individuals, and loci flagged as affected by potential genotyping error in more than 1% of individuals. At this point individuals that had not been genotyped for a minimum of 95% of loci were removed from the data set as well.

## Linkage map construction

For each mapping family, *r/qtl* [12] was used to further filter the data set and create male and female-specific linkage maps; *onemap* [13] was then used to generate a family map based on full-sibling genotypes. The two family maps were merged, using *LPmerge* [14], to create a consensus map. The detailed steps are documented at in reproducible R notebooks at: https://github.com/sjoleary/SFL_LinkageMap/blob/master/LinkageMapping_FamA.Rmd, https://github.com/sjoleary/SFL_LinkageMap/blob/master/LinkageMapping_FamB.Rmd, and https://github.com/sjoleary/SFL_LinkageMap/blob/master/MergeMaps.Rmd.

For each of the four sex-specific maps, a series of pre-mapping filtering steps were undertaken, using *r/qtl*, to omit duplicate individuals and loci with distorted segregation patterns. For groups of loci that segregated together (i.e., had identical segregation patterns), only one locus was retained for mapping. Recombination fractions (rf) and LOD scores were calculated for each pairwise marker combination and markers were grouped into linkage groups based in a minimum LOD = 6 and a maximum rf = 0.35. An initial marker order was established using a greedy algorithm which adds one marker at a time with the previously added marker locations fixed. Final positions are determined by minimizing the number of obligate crossovers.

After initial ordering, the quality of the marker order was assessed by identifying large gaps between loci, indicating that adjacent markers were weakly linked, and by dropping markers one by one for each linkage group to identify problematic markers (indicated by a decrease in estimated chromosome length and an increase in LOD). Next, the number of crossovers per individuals was determined and individuals with >50 crossovers removed [12]. Finally, genotyping error was determined by calculating the LOD score for each genotype and measuring evidence for genotyping error according to Lincoln & Lander ([15]; genotypes with an error LOD >4 and genotyping errors indicated by tight double-crossovers were identified and coded as missing. After removing problematic loci and individuals, markers were re-ordered and a sliding window was used to compare alternate marker orders based on the number of obligate crossovers and likelihood scores. Marker order was switched to the order with the lowest number of crossovers, highest likelihood, and shortest chromosomes. The final marker order was again assessed to identify problematic markers based on weak linkage, dropping markers one by one and assessing tight crossovers. Finally, sex-specific maps were finalized by assigning all loci from co-segregating groups to the same location as the appropriate mapped locus.

After both the male- and female maps were created, they were compared to identify the number of shared loci per map. Markers with identical segregation patterns across sexes were temporarily removed from the data set during the mapping process. Two-point recombination fractions were calculated between all pairs of markers [16], using *onemap*, which implements algorithms for simultaneous maximum-likelihood estimation of linkage and linkage phase in a full-cross data set. Markers were assigned to linkage groups based on a minimum LOD = 6 and a maximum rf = 0.35, and ordered using an initial subset of the most informative markers; the remaining markers were mapped by estimating parameters for all possible maps and placing markers in the most likely position. Alternative marker orders were compared using a ripple with a window size = 4. Family maps were finalized by assigning all loci from co-segregating groups to the same location as the appropriate mapped locus.

Family maps were checked for quality of marker order (as above) and the two maps compared to identify incongruent marker orders. The location of problematic markers was identified on both family maps to determine if they were a part of a cluster of loci of zero observed recombination. Problematic markers were removed from the map in which their location was not resolved and markers then re-ordered and mapped. This process was repeated three times to ensure the best possible mapping given the constraints of sample size and possible genotyping error. Once all problematic markers were removed, *LPmerge* was used to create a consensus linkage map by merging corresponding linkage groups from mapping cross A and B. *LPmerge* uses linear programming to minimize mean absolute error between the consensus map and individual family maps. The algorithm uses linear inequality restraints to ensure that marker order in the consensus map consistently reflects the order in the merged maps. *LPmerge* was run for a bin size K = 1 – 100 and the consensus map chosen based on minimizing the root mean-squared error (RMSE) between the consensus and individual linkage maps.

## Comparative genomics and synteny mapping

The *synteny_mapper* pipeline [17] was used to determine patterns of synteny between the consensus linkage map and six fully sequenced fish genomes, European seabass, *Dicentrarchus labrax*, barramundi, *Lates calcarifer*, three-spined stickleback, *Gasterosteus aculatus*, Nile tilapia *Oreochromis niloticus*, fugu, *Takifugu rubripes*, and green spotted puffer, *Tetraodon nigroviridis*, following Hollenbeck et al. [18]. This pipeline blasts loci mapped in the linkage map to fully sequenced fish genomes and determines their relative positions on the corresponding chromosomes in order to identify syntenic blocks [18,19]. Order mismatches separated by less than 5% of the total length of the linkage group were considered the result of either small-scale local arrangements or ordering errors due to inherent uncertainty in the mapping process and were ignored in the process of identifying syntenic blocks.

Two data sets from gene expression studies in European flounder, composed of genes differentially expressed between habitats with marked differences in salinity and/or up- and down-regulated as a response to differences in salinity [20], and exposure to a variety of pollutants [21] in wild and lab populations were downloaded, as were two data sets of microsatellite loci used to create a linkage map for Japanese flounder and for QTL analyses [22–25]. Using the *synteny_mapper* pipeline these sequences were then synteny mapped onto the consensus southern flounder linkage map as outline in [18]. In short, transcripts are blasted to the previously mentioned six fully assembled and annotated fish genome. If transcripts mapped to a syntenic block, they were located on the linkage map by identifying the SNP-containing locus to the left and right within the synteny block.

**References:**

1. Portnoy DS, Puritz JB, Hollenbeck CM, Gelsleichter J, Chapman D, Gold JR. Selection and sex-biased dispersal: the influence of philopatry on adaptive variation. PeerJ. 2015;1–20.

2. Catchen JM, Amores A, Hohenlohe P, Cresko W, Postlethwait JH. Stacks : Building and Genotyping Loci De Novo From Short-Read Sequences. G3 Genes|Genomes|Genetics. 2011;1:171–82.

3. Puritz JB, Hollenbeck CM, Gold JR. dDocent : a RADseq, variant-calling pipeline designed for population genomics of non-model organisms. PeerJ. 2014;2:e431.

4. Fu L, Niu B, Zhu Z, Wu S, Li W. CD-HIT: Accelerated for clustering the next-generation sequencing data. Bioinformatics. 2012;28:3150–2.

5. Zhang J, Kobert K, Flouri T, Stamatakis A. PEAR: A fast and accurate Illumina Paired-End reAd mergeR. Bioinformatics. 2014;30:614–20.

6. Ilut DC, Nydam ML, Hare MP. Defining loci in restriction-based reduced representation genomic data from nonmodel species: Sources of bias and diagnostics for optimal clustering. Biomed Res. Int. 2014;2014.

7. Li H, Durbin R. Fast and accurate short read alignment with Burrows-Wheeler transform. Bioinformatics. 2009;25:1754–60.

8. Garrison E, Marth G. Haplotype-based variant detection from short-read sequencing. PLoS One. 2012;11:e0151651.

9. Danecek P, Auton A, Abecasis G, Albers CA, Banks E, DePristo MA, et al. The variant call format and VCFtools. Bioinformatics. 2011;27:2156–8.

10. vcfallelelicprimitives. Available from: https://github.com/vcflib/vcflib#vcflib

11. rad_haplotyper [Internet]. Available from: https://github.com/chollenbeck/rad_haplotyper

12. Broman KW, Wu H, Sen Ś, Churchill GA. R/qtl: QTL mapping in experimental crosses. Bioinformatics. 2003;19:889–90.

13. Margarido GRA, Souza AP, Garcia AAF. OneMap: software for genetic mapping in outcrossing species. Hereditas. 2007;144:78–9.

14. Endelman JB, Plomion C. LPmerge: An R package for merging genetic maps by linear programming. Bioinformatics. 2014;30:1623–4.

15. Lincoln SE, Lander ES. Systematic detection of errors in genetic linkage data. Genomics. 1992;14:604–10.

16. Wu R, Ma C-X, Painter I, Zeng Z-B. Simultaneous Maximum Likelihood Estimation of Linkage and Linkage Phases in Outcrossing Species. Theor. Popul. Biol. 2002;61:349–63.

17. syteny_mapper. Available from: https://github.com/chollenbeck/synteny_mapper

18. Hollenbeck CM, Portnoy DS, Gold JR. A genetic linkage map of red drum (Sciaenops ocellatus) and comparison of chromosomal syntenies with four other fish species. Aquaculture. 2015;435:265–74.

19. Hollenbeck CM, Portnoy DS, Wetzel D, Sherwood TA, Samollow PB, Gold JR. Linkage mapping and comparative genomics of red drum (Sciaenops ocellatus) using next-generation sequencing. G3 Genes Genomes Genet. 2017;7:843–50.

20. Larsen PF, Nielsen EE, Williams TD, Hemmer-Hansen J, Chipman JK, Kruhoffer M, et al. Adaptive differences in gene expression in European flounder (Platichthys flesus). Mol. Ecol. 2007;16:4674–83.

21. Falciani F, Diab AM, Sabine V, Williams TD, Ortega F, George SG, et al. Hepatic transcriptomic profiles of European flounder (Platichthys flesus) from field sites and computational approaches to predict site from stress gene responses following exposure to model toxicants. Aquat. Toxicol. 2008;90:92–101.

22. Castaño-Sánchez C, Fuji K, Ozaki A, Hasegawa O, Sakamoto T, Morishima K, et al. A second generation genetic linkage map of Japanese flounder (Paralichthys olivaceus). BMC Genomics. BioMed Central Ltd; 2010;11:554.

23. Song W, Pang R, Niu Y, Gao F, Zhao Y, Zhang J, et al. Construction of High-Density Genetic Linkage Maps and Mapping of Growth-Related Quantitative Trail Loci in the Japanese Flounder (Paralichthys olivaceus). PLoS One. 2012;7:e50404.

24. Wang X, Xu W, Liu Y, Wang L, Sun H, Wang L, et al. Quantitative trait loci detection of Edwardsiella tarda resistance in Japanese flounder Paralichthys olivaceus using bulked segregant analysis. Chinese J. Oceanol. Limnol. Science Press; 2016;34:1297–308.

25. Fuji K, Kobayashi K, Hasegawa O, Coimbra MRM, Sakamoto T, Okamoto N. Identification of a single major genetic locus controlling the resistance to lymphocystis disease in Japanese flounder (Paralichthys olivaceus). Aquaculture. 2006;254:203–10.
