# Supplementary material for: Genetic mapping and comparative genomics to inform restoration enhancement and culture of southern flounder, Paralichthys lethostigma
Source: BMC Genomics. 2018 Feb 23;19:163. doi: 10.1186/s12864-018-4541-0 (PMC5824557; doi:10.1186/s12864-018-4541-0)
Supplement: Supplementary file 2 — Filtering parameters for each filtering step, and number of SNPs and contigs remaining for Families A and B. (DOCX 15 kb) [file 12864_2018_4541_MOESM2_ESM.docx]

**Additional File 2:** Filtering parameters for each filtering step, and number of SNPs and contigs remaining for Families A and B.

| **Filter** | **Parameter** | **SNPs (Fam A)** | **Contigs (Fam A)** | **SNPs (Fam B)** | **Contigs (Fam B)** |
| --- | --- | --- | --- | --- | --- |
| Raw SNPs |  | 178,644 | 39,078 | 447,144 | 39,950 |
| Filter 0 | minQ >20 | 141,881 | 37,233 | 281,853 | 39,217 |
| Filter 1 | mac > 3, geno > 0.5 | 65,259 | 19,670 | 169,426 | 28,335 |
| Filter 2 | min DP > 3, geno > 0.5 | 32,355 | 9,286 | 47,027 | 9,409 |
| Filter 3 | geno > 0.6, max-missing ind < 0.9 | 27,905 | 7,874 | 30,217 | 6,579 |
| Filter 4 | maf > 0.01, min-mean DP 5 | 26,843 | 7,546 | 25,523 | 5,950 |
| Filter 5 | geno 0.8, max-missing ind < 0.75 | 20,578 | 5,814 | 23,147 | 5,449 |
| Filter 6 | maf < 0.03, min-mean DP 10 (20) | 20,258 | 5,791 | 20,919 | 5,152 |
| Filter 7 | geno 0.85, max-missing ind > 65% | 18,277 | 5,222 | 19,132 | 4,755 |
| Filter 8a | Allelic balance | 17,262 | 5,157 | 18,076 | 4,705 |
| Filter 8b | Ratio quality/depth, | 16,932 | 5,132 | 17,678 | 4,683 |
| Filter 8c | Ratio mapping quality | 16,833 | 5,119 | 17,635 | 4,679 |
| Filter 9 | Loci with reads from both strands | 16,546 | 5,063 | 17,512 | 4,654 |
| Filter 10 | Properly paired status | 16,471 | 5,038 | 17,439 | 4,629 |
| Filter 11 | Maximum depth | 16,421 | 5,026 | 16,638 | 4,450 |
| Filter 12 | Remove indels | 16,680 | 4,892 | 16,805 | 4,357 |
| Filter 13 | maf 0.025 (0.05) | 16,674 | 4,892 | 16,802 | 4,357 |
| Filter 14 | max-missing 0.95 | 15,180 | 4,461 | 16,802 | 4,357 |
| Filtered data set |  | 15,180 | 4,461 | 16,802 | 4,357 |
| Haplotyped data set |  |  | 2,773 |  | 2,353 |

Min Q: minimum quality

Mac: minor allele count

Geno: genotype call rate (per locus)

Min DP: minimum depth (per genotype)

Max-missing ind: maximum allowed missing data per individual

Min-mean DP: minimum mean depth per locus (across all individuals)

Maf: minor allele frequency
