# Supplementary material for: Genetic mapping and comparative genomics to inform restoration enhancement and culture of southern flounder, Paralichthys lethostigma
Source: BMC Genomics. 2018 Feb 23;19:163. doi: 10.1186/s12864-018-4541-0 (PMC5824557; doi:10.1186/s12864-018-4541-0)
Supplement: Supplementary file 4 — Comparison of number of loci and linkage group length (in parentheses) for corresponding linkage groups for consensus map (LG), family maps (LG_Fam A, LG_Fam B), and sex-specific maps (LG_Fam A_F, LG_FamA_M, LG_FamB_A, LG_FamB_M). (DOCX 16 kb) [file 12864_2018_4541_MOESM4_ESM.docx]

**Additional File 3: Comparison of number of loci and linkage group length** (in parentheses) for corresponding linkage groups for consensus map (LG), family maps (LG_Fam A, LG_Fam B), and sex-specific maps (LG_Fam A_F, LG_FamA_M, LG_FamB_A, LG_FamB_M).

| **LG** | **LG_FamA** | **LG_FamA_F** | **LG_FamA_M** | **LG_FamB** | **LG_FamB_F** | **LG_FamB_M** |
| --- | --- | --- | --- | --- | --- | --- |
| 1 (161/64.64) | 1 (90/66.083) | 1 (81/61.036) | 1 (75/62.607) | 1 (95/57.195) | 1 (78/57.489) | 1 (80/54.059) |
| 2 (118/70.79) | 2 (73/50.382) | 3 (64/62.829) | 13 (45/93.543) | 15 (58/59.838) | 17 (42/40.596) | 20 (39/63.93) |
| 3 (109/71.05) | 3 (58/33.478) | 5 (62/54.055) | 19 (55/64.16) | 8 (60/67.932) | 8 (47/58.908) | 11 (49/51.165) |
| 4 (126/74.35) | 4 (69/68.086) | 6 (57/61.175) | 14 (51/63.471) | 2 (72/72.717) | 2 (53/50.054) | 3 (68/95.88) |
| 5 (136/75.92) | 5 (82/66.74) | 7 (58/53.941) | 8 (62/63.862) | 4 (71/57.047) | 4 (58/23.593) | 2 (64/55.715) |
| 6 (135/64.34) | 6 (81/66.314) | 8 (62/59.348) | 6 (66/83.523) | 3 (68/75.618) | 3 (57/64.522) | 13 (44/78.966) |
| 7 (120/64.35) | 7 (78/53.611) | 9 (55/47.252) | 9 (67/56.465) | 10 (52/51.883) | 11 (48/25.813) | 24 (20/49.622) |
| 8 (130/60.79) | 8 (77/68.3) | 10 (69/59.024) | 10 (60/77.373) | 12 (65/72.571) | 13 (56/68.802) | 12 (38/32.433) |
| 9 (129/68.23) | 9 (70/57.077) | 12 (52/54.599) | 12 (58/48.529) | 6 (72/78.519) | 6 (46/32.167) | 4 (64/98.769) |
| 10 (118/65.97) | 10 (67/54.589) | 13 (50/48.785) | 7 (41/118.869) | 21 (59/61.911) | 23 (24/45.975) | 18 (44/61.141) |
| 11 (112/78.52) | 11 (69/57.902) | 15 (60/56.22) | 11 (52/65.39) | 24 (56/61.546) | 16 (46/60.406) | 15 (49/56.417) |
| 12 (136/65.44) | 12 (80/64.117) | 16 (52/51.465) | 2 (63/61.158) | 5 (66/64.579) | 5 (52/37.156) | 6 (37/89.957) |
| 13 (111/57.13) | 13 (67/57.911) | 17 (50/52.746) | 20 (53/61.612) | 11 (58/64.352) | 12 (46/60.805) | 17 (40/44.759) |
| 14 (97/67.93) | 14 (64/53.196) | 19 (51/51.593) | 15 (47/64.47) | 22 (38/51.805) | 24 (19/42.033) | 23 (31/49.564) |
| 15 (101/66.74) | 15 (58/53.673) | 20 (49/54.88) | 18 (47/50.105) | 16 (51/75.186) | 18 (42/62.339) | 19 (38/52.578) |
| 16 (117/65.87) | 16 (67/48.133) | 21 (57/45.936) | 16 (52/48.379) | 14 (62/70.578) | 15 (40/57.165) | 7 (51/60.728) |
| 17 (89/56.3) | 17 (52/61.422) | 22 (42/56.945) | 24 (35/71.71) | 18 (45/58.789) | 20 (34/53.164) | 22 (33/70.291) |
| 18 (112/72.57) | 18 (54/56.173) | 23 (43/57.487) | 21 (44/54.899) | 17 (67/76.593) | 19 (47/53.61) | 9 (47/68.162) |
| 19 (99/76.28) | 19 (56/47.848) | 14 (50/50.228) | 17 (45/43.268) | 13 (51/56.784) | 14 (41/37.896) | 16 (41/24.422) |
| 20 (132/71.55) | 20 (72/60.947) | 11 (50/62.693) | 22 (58/58.416) | 7 (70/71.508) | 7 (53/39.907) | 10 (47/59.097) |
| 21 (71/65.46) | 21 (39/34.695) | 24 (31/33.542) | 23 (33/48.057) | 19 (42/64.341) | 21 (34/29.145) | 21 (36/52.274) |
| 22 (121/53.2) | 22 (71/55.471) | 18 (57/90.41) | 5 (53/60.547) | 9 (61/75.918) | 10 (52/59.906) | 5 (46/53.842) |
| 23 (135/70) | 23 (85/63.287) | 4 (64/122.617) | 3 (64/61.923) | 20 (61/64.117) | 22 (25/57.068) | 8 (52/57.939) |
| 24 (132/58.01) | 24 (93/55.786) | 2 (39/120.677) | 4 (77/57.531) | 23 (53/58.526) | 9 (47/56.992) | 14 (20/53.848) |
