# Supplementary figures and images for: Genetic mapping and comparative genomics to inform restoration enhancement and culture of southern flounder, Paralichthys lethostigma
Source: BMC Genomics. 2018 Feb 23;19:163. doi: 10.1186/s12864-018-4541-0 (PMC5824557; doi:10.1186/s12864-018-4541-0)

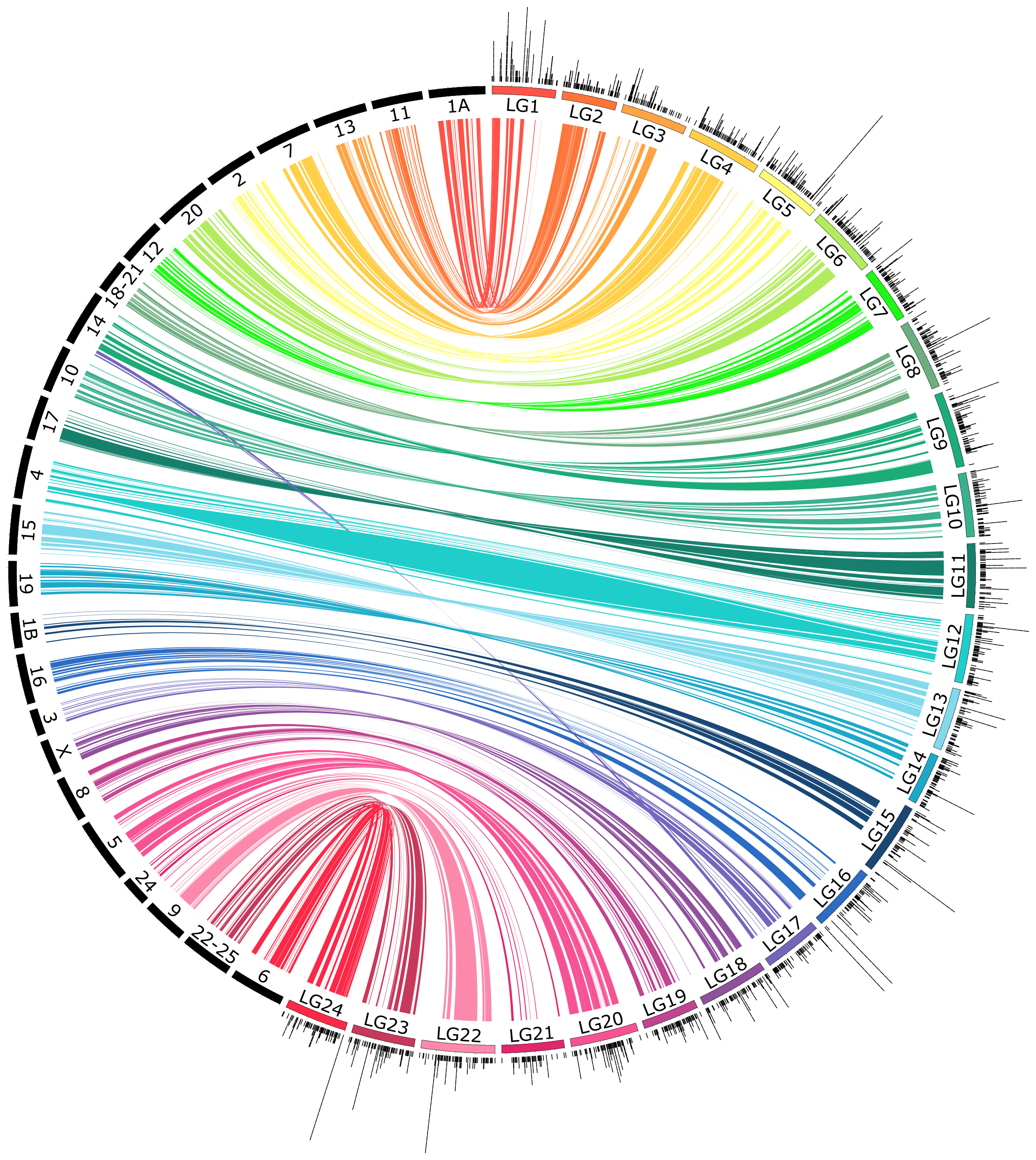

Supplement: Supplementary file 5 — Comparative view of location of syntenic blocks on consensus linkage map of southern flounder and European seabass. Solid black rectangles represent chromosomes of European seabass. Black ticks indicate the positions of loci mapped on southern flounder linkage groups (colored rectangles); loci mapped to the same location are stacked. Syntenic blocks are connected by ribbons; the color corresponds to the color of each linkage group. Width of the ribbon represents size of the syntenic block on a linkage group and its corresponding location on the chromosome of each comparison species. (PNG 3116 kb) [file 12864_2018_4541_MOESM5_ESM.png]

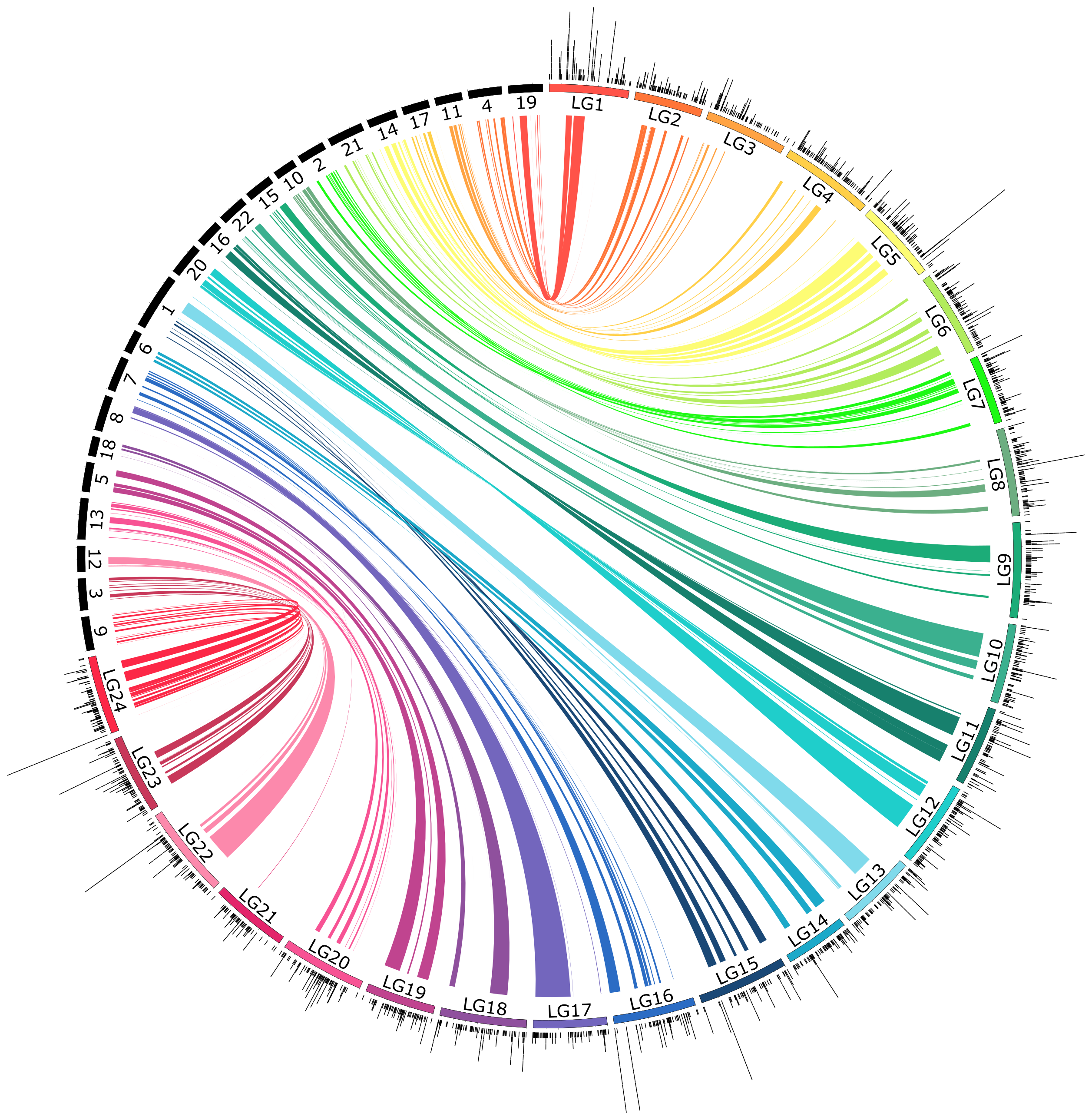

Supplement: Supplementary file 6 — Comparative view of location of syntenic blocks on consensus linkage map of southern flounder and fugu. Solid black rectangles represent chromosomes of fugu. Black ticks indicate the positions of loci mapped on southern flounder linkage groups (colored rectangles); loci mapped to the same location are stacked. Syntenic blocks are connected by ribbons; the color corresponds to the color of each linkage group. Width of the ribbon represents size of the syntenic block on a linkage group and its corresponding location on the chromosome of each comparison species. (PNG 2296 kb) [file 12864_2018_4541_MOESM6_ESM.png]

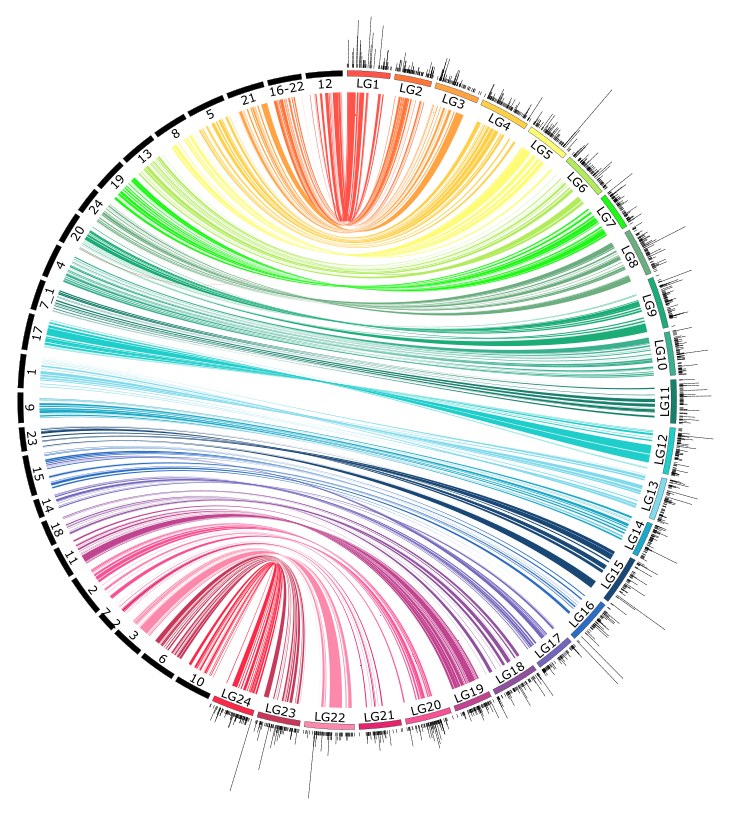

Supplement: Supplementary file 7 — Comparative view of location of syntenic blocks on consensus linkage map of southern flounder and barramundi. Solid black rectangles represent chromosomes of barramundi. Black ticks indicate the positions of loci mapped on southern flounder linkage groups (colored rectangles); loci mapped to the same location are stacked. Syntenic blocks are connected by ribbons; the color corresponds to the color of each linkage group. Width of the ribbon represents size of the syntenic block on a linkage group and its corresponding location on the chromosome of each comparison species. (PNG 584 kb) [file 12864_2018_4541_MOESM7_ESM.png]

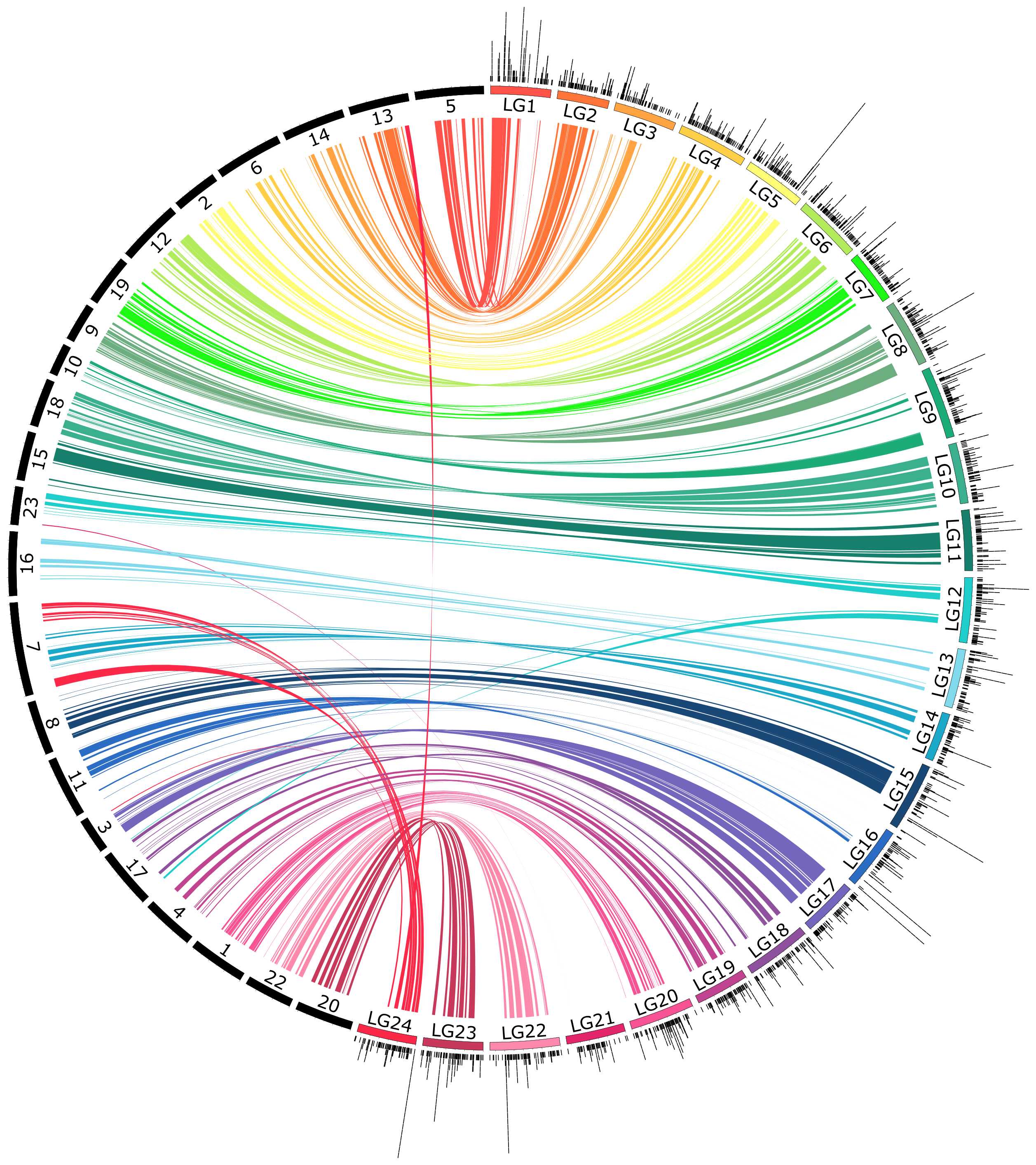

Supplement: Supplementary file 8 — Comparative view of location of syntenic blocks on consensus linkage map of southern flounder and nile tilapia. Solid black rectangles represent chromosomes of nile tilapia. Black ticks indicate the positions of loci mapped on southern flounder linkage groups (colored rectangles); loci mapped to the same location are stacked. Syntenic blocks are connected by ribbons; the color corresponds to the color of each linkage group. Width of the ribbon represents size of the syntenic block on a linkage group and its corresponding location on the chromosome of each comparison species. (PNG 2546 kb) [file 12864_2018_4541_MOESM8_ESM.png]

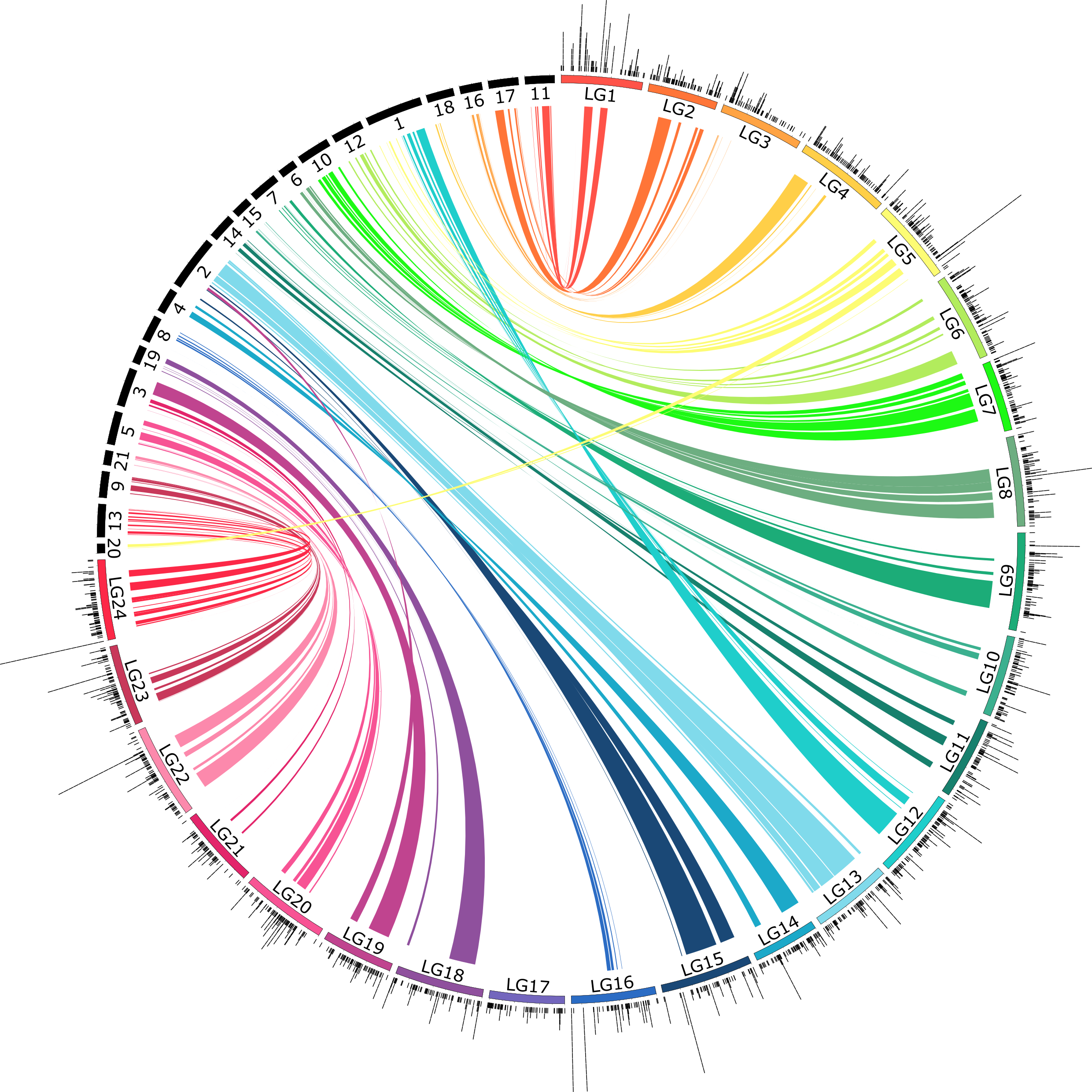

Supplement: Supplementary file 9 — Comparative view of location of syntenic blocks on consensus linkage map of southern flounder and green spotted puffer. Solid black rectangles represent chromosomes of green spotted puffer. Black ticks indicate the positions of loci mapped on southern flounder linkage groups (colored rectangles); loci mapped to the same location are stacked. Syntenic blocks are connected by ribbons; the color corresponds to the color of each linkage group. Width of the ribbon represents size of the syntenic block on a linkage group and its corresponding location on the chromosome of each comparison species. (PNG 1925 kb) [file 12864_2018_4541_MOESM9_ESM.png]
